# Clinic Follow up and Neurological Disability in Children Following Pregnancies Complicated by Preterm Rupture of Membranes and Preeclampsia

**Published:** 2021-05-31

**Authors:** Laura Paulson, Dianne Thornhill, Jennifer Armstrong

**Affiliations:** 1 School of Public Health, University of Colorado Anschutz School of Medicine, Aurora, Colorado, USA; 2 Hemophilia and Thrombosis Center, University of Colorado Anschutz School of Medicine, Aurora, Colorado, USA; 3 Department of Pediatrics, Section of Neurology, University of Colorado Anschutz School of Medicine, Aurora, Colorado, USA; 4 Department of OB/GYN, University of Colorado Anschutz School of Medicine, Aurora, Colorado, USA

**Keywords:** Cerebral palsy, Language, Sensorimotor, Race/ethnicity, Outcomes, Neurodevelopment

## Abstract

**Context:**

Preeclampsia and preterm premature rupture of membranes (PPROM) have been associated with perinatal brain injury. Despite a strong understanding of the relationships between preterm birth and neurologic deficits, and between PPROM and preeclampsia and preterm birth, the relationship between PPROM and preeclampsia and neurologic disability is not well characterized.

**Objective:**

We compared trends in neurologic deficits in children born to mothers with these conditions and described differences in patient characteristics among follow up visit attendance.

**Methods:**

We conducted a prospective cohort study of women with preeclampsia or PPROM. Neurologic deficits were assessed with the Pediatric Stroke Outcome Measure at follow up visits through age 10 years. Eighty nine of the 178 women enrolled completed at least one follow up. Results: Among children born >32 weeks, PPROM showed higher left and right sided sensorimotor deficits at initial follow (p=0.045, p=0.01). In children born ≤ 32 weeks, preeclampsia had higher language production deficits at 3 year follow up (p=0.05).

Sensorimotor deficits were greater and sustained in PPROM. Language production deficits were predominant among after 2 years of age in preeclampsia. Racial disparities were found in clinic attendance rates, with Black families most affected.

**Conclusion:**

Differences in neurodevelopmental patterns suggest differences in underlying neuronal injuries. Neurologic assessment should occur routinely throughout early childhood to detect delayed deficits after PPROM and preeclampsia and ensure inclusion of underserved or at risk populations.

## Introduction

Preeclampsia and preterm premature rupture of membranes are two increasingly common complications of pregnancy which are both associated with high rates of preterm birth. Preeclampsia is characterized by hypertension and signs of damage to another organ system after 20 weeks gestation in women who have otherwise had normal blood pressure. It is estimated to affect 3.8% of pregnancies in the United States and 2%−8% of pregnancies worldwide, and is estimated to cause 15% of preterm births [1]. PPROM is defined as the breakage of the amniotic sac before 34 weeks gestation. It is estimated to affect 3% of pregnancies and cause a third of preterm births [2].

The relationship between preterm birth and perinatal brain injury has been well documented [3]. Due to advances in obstetric interventions, babies are being born earlier [3,4] thus increasing opportunity for the complications associated with preterm birth, such as neurologic disabilities. Perinatal brain injury has been associated with substantial risk of long term disability [5]. The most severe injuries can lead to life-long issues such as cerebral palsy as well as cognitive and behavioral impairments and impaired motor outcomes. Prediction of long term outcomes after perinatal brain injury has generally proved difficult, although cognitive and neuromotor impairments at age 5 have been found to increase with decreasing gestational age among very preterm infants [3].

Despite a strong understanding of the relationships between preterm birth and neurologic deficits, and between PPROM and preeclampsia and preterm birth, the relationship between PPROM and preeclampsia and neurologic disability is not well characterized.

Research in long term neurodevelopmental outcomes has typically excluded patients with PPROM or preeclampsia. There is an opportunity to study a population that is clearly at risk. We sought to compare trends in neurologic deficits over time in children born to mothers with PPROM and preeclampsia and characterize potential characteristics among levels of follow up visit attendance.

## Methods

Pregnant women with PPROM or preterm preeclampsia (>24 or ≤ 34 weeks) who were hospitalized at University of Colorado Hospital between June 2010 and June 2016 were enrolled in a prospective cohort study. Patients were excluded if they were under 18 years of age, carrying a triplet or greater gestation pregnancy, or carrying a non-viable fetus or fetus with multiple congenital abnormalities. Demographic and pregnancy data were abstracted from the Perinatal Database of the Department of Obstetrics and Gynecology at the University Of Colorado School Of Medicine by a trained research assistant using a standardized protocol.

Offspring of these pregnancies were asked underwent standardized neurology exams at the Hemophilia and Thrombosis Center at the University of Colorado at designated age intervals. The Pediatric Stroke Outcome Measure (PSOM) was used to assess neurologic disability. This measure is widely used for pediatric stroke patients and has been validated in that population [6]. The PSOM provides a total score of neurologic deficits ranging from 0–10, with 0 representing no deficit and 10 representing maximum deficit. The total score is a composite of five different scales of neurologic functioning: sensorimotor deficits on the left side of the body, sensorimotor deficits on the right side of the body, deficits in language production, deficits in language comprehension, and cognitive behavioral deficits; scores for each of these scales range from 0–2.

### Statistics

Median PSOM scores were used because of the positively skewed distribution of scores, which is common in this measure [6]. Because gestational age at birth is highly correlated with neurological deficits, analyses were corrected for gestational age. In non-parametric tests, this correction was achieved by stratifying children into either >32 weeks or ≤ 32 weeks gestational age at birth. When comparing attendance at follow up assessments, patients were classified as having high attendance (≥ 70% attendance rate) or low attendance (≤ 30% attendance rate). These cut offs were chosen based on the distribution of attendance rates among all who attended at least one visit.

Group differences in PSOM scores were analyzed with Mann Whitney U tests. T tests were used for testing group differences in patient characteristics. Growth curve models, repeated measures ANOVA and Friedman tests were used to model longitudinal trends in PSOM scores. All analyses were conducted in SPSS Statistics 24, except for growth curves, which were modeled in MPlus Version 7. All tests were 2 sided and P<0.05 was considered significant.

## Results

There were 178 women initial enrolled in the study. Of those, 89 maternal child dyads completed at least one follow up visit. Maternal characteristics were similar between the PPROM and preeclamptic mothers, except that BMI was significantly higher in the preeclamptic mothers (34.96 vs 30.69, p=0.007). C section deliveries were significantly higher with preeclampsia (59.5% vs 36.2%, p<0.001). There was no difference in birth weight or sex of the newborn groups (Table 1).

**Figure 1:**
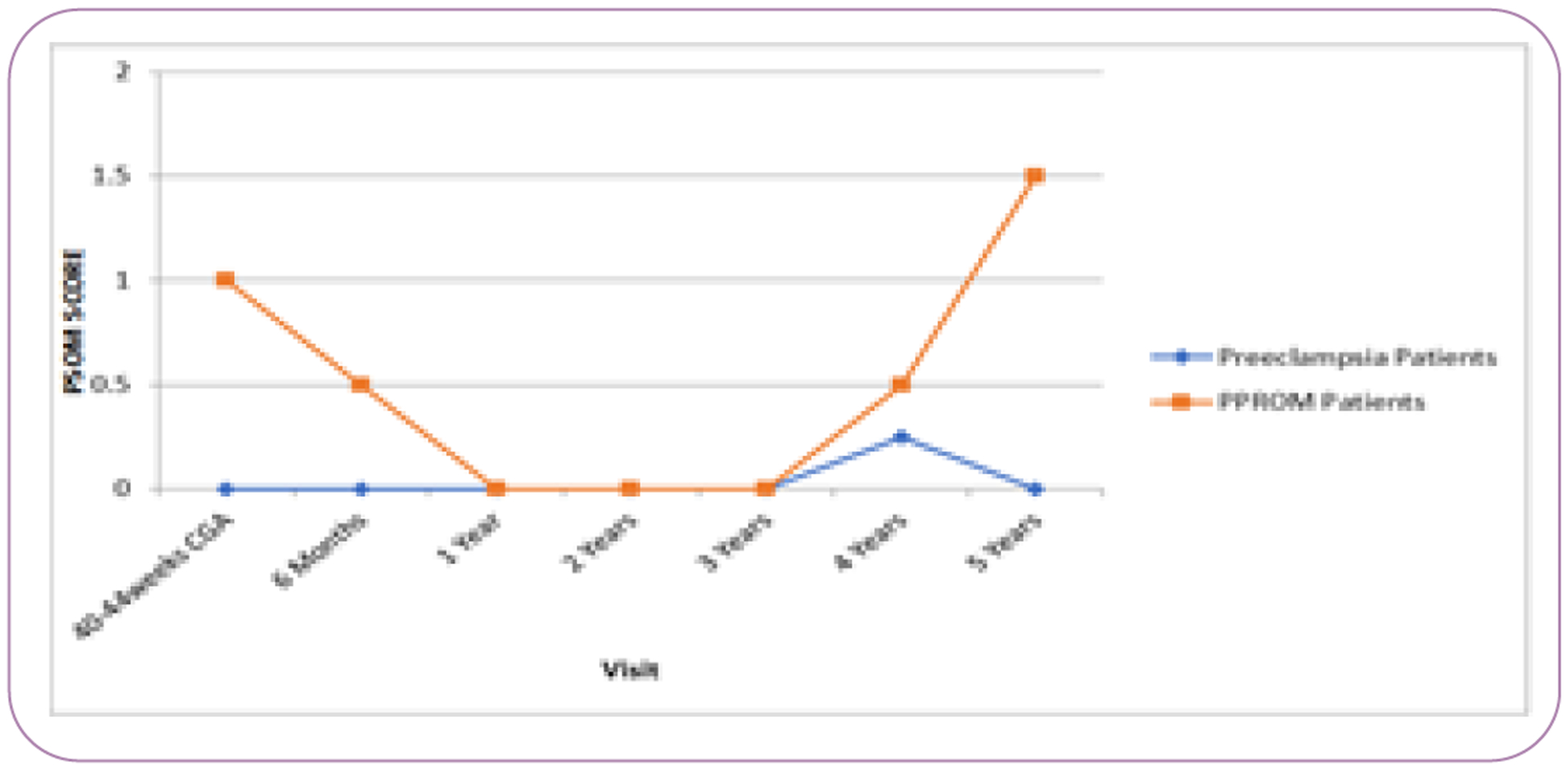
Median Pediatric Stroke Outcome Measure (PSOM) score by maternal risk factor. PPROM – Preterm Premature Rupture of Membranes; CGA – corrected gestational age

Overall, PPROM children tended to have worse PSOM scores than preeclampsia even after correcting for gestational age at birth (Figure 1). PSOM score was significantly higher at corrected term gestational age (p=0.0012), with the PPROM group having worse scores (median PSOM=1 versus 0). When looking at developmental subcategories, there were worse sensorimotor deficits on both the right and left side of the body with PPROM (p=0.04; p=0.005, respectively). When analyzed by gestational age, PPROM infants born at >32 weeks gestational age had worse sensorimotor deficits at corrected term age compared to preeclampsia infants born at >32 weeks (p=0.01), but no difference at if born at <32 weeks. There were no significant differences in the other subcategories at correct term gestational age.

**Figure 2:**
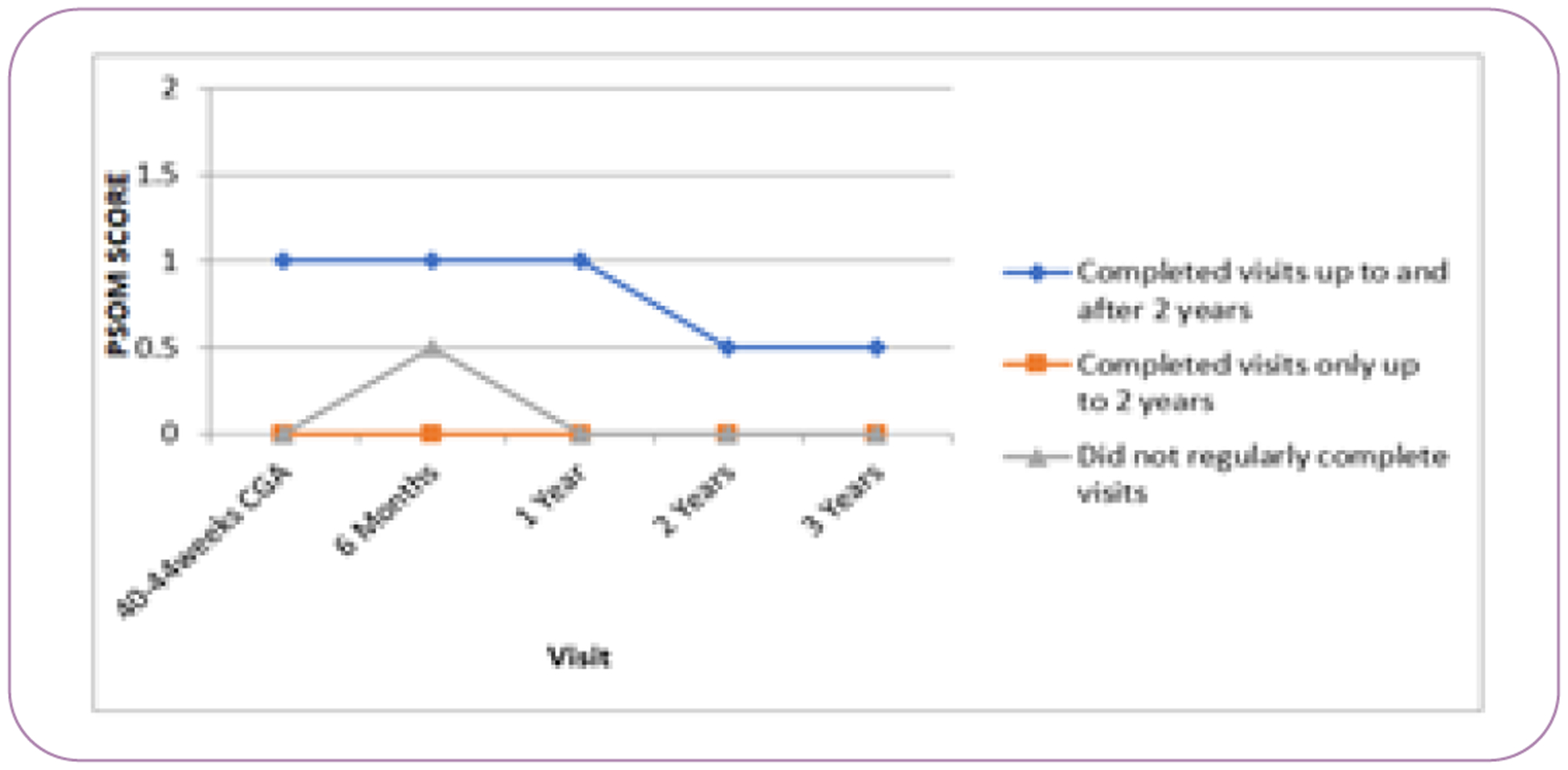
Median Pediatric Stroke Outcome Measure (PSOM) score, but clinic attendance before and after 2 years. CGA – corrected gestational age

**Figure 3:**
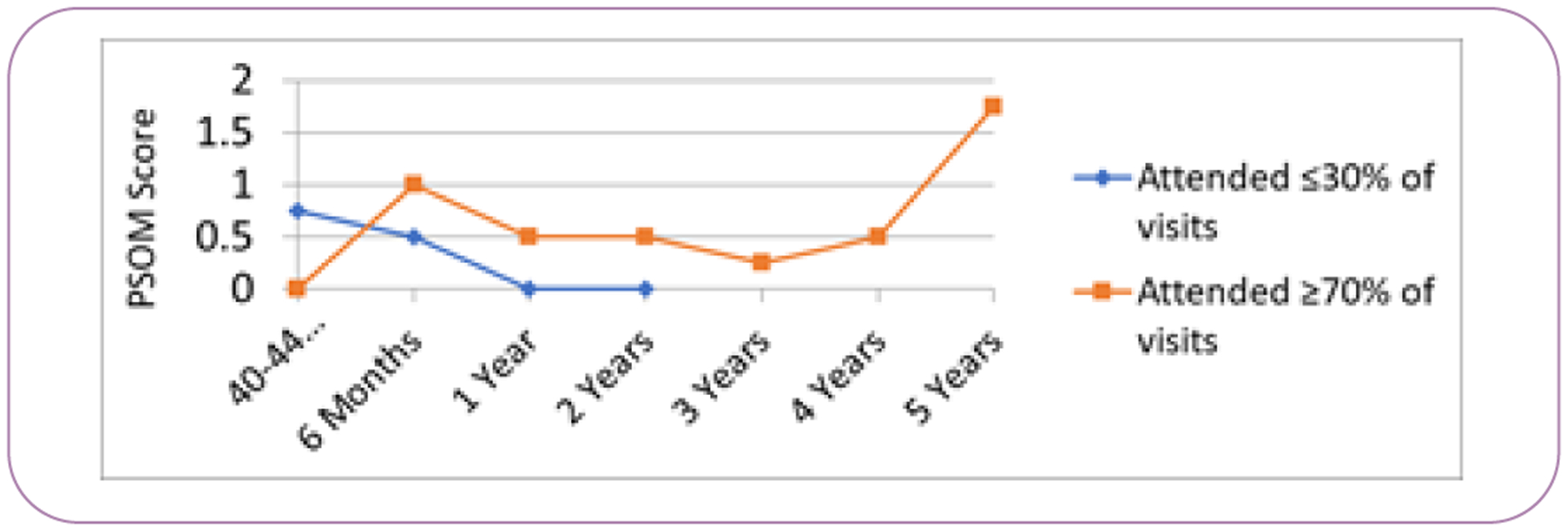
Median Pediatric Stroke Outcome Measure (PSOM) score by clinic attendance rate. CGA – Corrected gestational age.

There were notable disparities over time with visit follow up. Although African American/Black mothers accounted for less than 4% of the overall participants, they disproportionately represented the highest percentage lost to follow up 19.4% (Table 2). Follow up waned over time, with the majority completing visits through 3 years, but only 8/89 (<1%) of the children attended follow up visits at 5 years. PPROM and preeclampsia were equally represented at follow up visits. Of those that completed 5 year visit, the majority were non-Hispanic white mothers (62.5%). PSOM scores were generally worse in those who continued to come for follow up and typical development of those who did not return (Figures 2 and 3).

Higher/concerning PSOM score at 6 months was a significant independent predictor of both attendance and elevated PSOM score at 5 years (p=0007).

**Figure 4:**
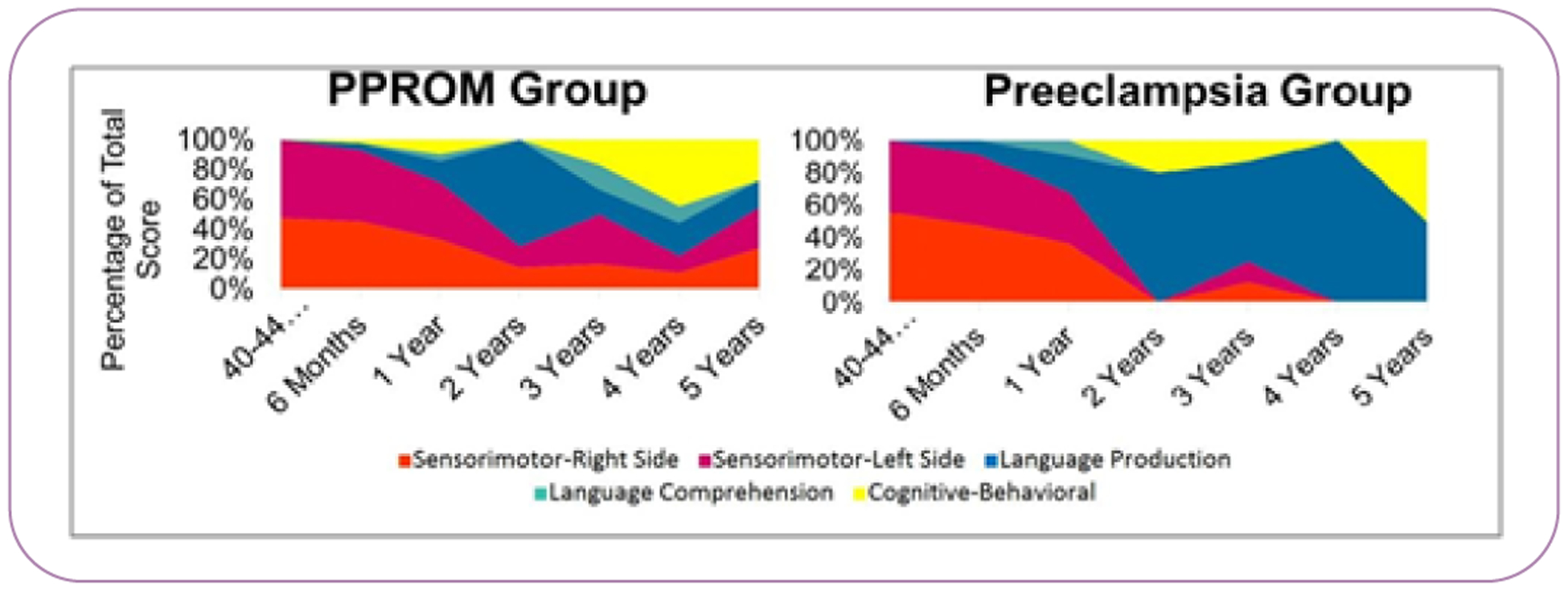
Percentages of PSOM total score associated with disability type, by maternal risk factor. PSOM – Pediatric Stroke Outcome Measure; PPROM – preterm premature rupture of membranes

Applying PSOM score over time modeling, distinct patterns emerged between the PPROM and preeclampsia group (Figure 4). Although among all children, sensorimotor and language production deficits had significant change over time (p=0.001; p=0.002, respectively), the PSOM group tended to have continued sensorimotor concerns compared to the preeclampsia group, while language production concerns developed as the preeclampsia children aged. Notably language production deficits emerged at 3 years old in preeclampsia children born at ≤ 32 weeks compared to 3 year old PPROM children born at ≤ 32 weeks (p=0.05). Language comprehension and cognitive behavioral deficits were stable among the groups over time, although in both groups cognitive behavioral concerns became more apparent with age. Sensorimotor deficits at early visits were not highly correlated with language production or cognitive/behavioral deficits at later visits.

## Discussion

Differences in neurodevelopmental patterns between PPROM and preeclampsia groups indicate potential differences in underlying neurologic injury. Although differences were not consistently statistically significant, the findings that children born to mothers in the PPROM group had higher scores of neurologic disability than children born to mothers with preeclampsia, and the fact children in the PPROM group were significantly younger supports previous findings that decreased gestational age is associated with an increase in neurologic deficits [3]. This is evident in the types of differences seen in scores; sensorimotor deficits (more dominant among those in the PPROM group) could potentially involve multiple regions of the central and peripheral nervous systems, whereas language deficits (more dominant among those in the preeclampsia group) indicate a more focused injury in only speech areas of the brain.

Additionally, early deficits do not necessarily relate to deficits seen in later years. Even after early sensorimotor deficits resolve, there is continued risk for other deficits, most notably in verbal language. There is evidence from these analyses that even after sensorimotor deficits resolve or diminish early in development, there is still risk for language and cognitive behavioral deficits after age

This was most evident in those children born to mothers with preeclampsia, as seen in how proportions of deficit changed as children aged. Furthermore, low correlations between sensorimotor deficits at 40–44 weeks and 6 months visits with language and cognitive deficits at 3 years through 5 years visits also demonstrate that initial deficits may not predict later deficits. This is consistent with previous research that some effects of perinatal brain injury may be delayed [3,5,7]. Since most NICU graduate are not followed by a neurologist or developmental pediatrician consistently beyond 3 years if early infant concerns are not present, there is a potential gap in recognizing subtle language, cognitive/behavioral, and learning disabilities later, with having an unremarkable neonatal screening ultrasound adding an additional false sense of security, as demonstrated in our previous work [8]. Our data support the conclusion that if patients do not regularly attend assessments throughout early childhood, opportunities for detection of delayed deficits, such as language and cognitive behavioral deficits, are decreased.

The decision to continue with optional neurological assessments could easily be driven by parents’ awareness of deficits in their children; however, we saw no consistent relationship between poor scores and attendance rates, possibly due to the small sample size and other unmeasured factors that were affecting the decision to come. When examining rates of follow up, race and ethnic differences emerged; racial disparities in attendance rates indicate gaps in care and detection for patients who are often the most at risk. Black women are at increased risk of PPROM and preeclampsia [1] and attended assessments in our study in smaller percentages than White families. A geospatial analysis of patients’ zip codes and how this relates to their attendance would also be an ancillary helpful to elucidate whether transportation, distance and/or socioeconomic factors pose barriers to attend follow up visits. Therefore, a successful program should encompass community outreach, home based programs and/or mobile clinic to help engage non-white populations.

It is possible that the consenting process for this study was problematic, and partly responsible for low post birth attendance rates. Pregnant women were approached during labor and delivery hospitalization for PPROM or preeclampsia, a very high stress time. The study team noted that, when contacted for follow ups, some mothers do not even remember agreeing to be part of the study. Approaching women at a different time and place, or with a consenting process that involves structured interviewing or re consent, might improve compliance with further research.

Lack of MRI imaging to confirm neurologic injury limits comparisons between these patients to other studies in perinatal brain injury, such pediatric stroke. It also means that even though patients in our sample seem to have some similar patterns in PSOM scores and deficit types to pediatric stroke patients, we cannot generalize results of other research to our patients. Expanded modalities including functional MRI may aid in more nuanced prognostications as clinical injury may not be visible on a gross level, but rather at a cellular level. Adding a more definitive diagnosis of injury would also allow for future case control designs.

In conjunction with neuroimaging, increasing our understanding of long term patterns in neurologic disabilities and overcoming barriers to care are crucial for better outcomes after PPROM and preeclampsia. Higher scores on the PSOM have been correlated with poorer outcomes in cognitive ability, problem behaviors, adaptive behaviors, and social participation, and it is an important tool in assessing long term outcomes in pediatric stroke patients [7]. However, it has not been validated in our population; including imaging to make comparisons with pediatric stroke would help ensure that this measure is also appropriate for children born to mothers with PPROM and preeclampsia. Moreover, although it has been demonstrated to be reliable and valid [6] and is ubiquitous in pediatric stroke research, there is some question about whether the PSOM alone is a sufficient measure of neurologic deficits, particularly cognitive and behavioral deficits [7]. Adding additional validated, reliable measures to create a battery would improve clinicians’ ability to accurately diagnose and predict long term outcomes in children born to mothers with these risk factors.

## Conclusion

Increasing our understanding of long term patterns in neurologic disabilities and overcoming barriers to care are crucial for better outcomes after PPROM and preeclampsia. The differences seen in patterns of deficit among children born to mothers with PPROM compared to those born to mothers with preeclampsia indicates a potential difference in underlying neurologic injury the children have sustained. Sample size limitation even within our best case scenario regional referral clinic illustrate the need for multicenter, collaborative research. Although many results were incomplete or not statistically significant because of the paucity of data in the sample, these analyses still contribute to establishing an evidence base for an at risk population that is not well characterized in existing literature. Ensuring that neurologic assessment occurs throughout early childhood to detect delayed deficits is an important finding from this research and has important public health implications. Educating families about long term risks and enlisting partners who are more likely to see patients, such as primary care doctors, could be an important step toward ensuring detection. Expansion of telehealth and improved access of internet and associated equipment may also be an important step in overcoming barriers to treatment such as lack of transportation. These analyses also provide guidance for future prospective studies of neurodevelopmental outcomes after PPROM and preeclampsia. Furthermore, since early intervention services end at age 3 years in most US states, it is important to recognize ongoing risk of neurodevelopmental disability and need for beyond toddlerhood including dedicated NICU graduate clinical programs and ongoing assessment language and cognitive behavioral therapy resources beyond toddlerhood.

## Figures and Tables

**Table 1:** Demographic characteristics. Means are expressed unless otherwise noted. PPROM – preterm premature rupture of membranes; VBAC – vaginal birth after cesarean delivery

	ALL PATIENTS	PPROM	PREECLAMPSIA
	(N=89)	(N=47)	(N=42)
Mother’s age at delivery (years)	29.28	30.3*	28.14*
Mother’s BMI at delivery	32.54	30.69*	34.96*
Gestational Age at delivery (weeks)	32.04	31.33*	32.82*
Baby’s Weight at Birth (kg)	1.78	1.76	1.81
Delivery Type-n (%)			
Vaginal	43 (48.3%)	26 (55.3%)	17 (40.5%)
C-Section	42 (47.2%)	17 (36.2%)	25 (59.5%)
VBAC	4 (4.5%)	4 (8.5%)	0
Mother’s Race- n (%)			
White/Caucasian	81 (91%)	41 (87.2%)	40 (95.2%)
Black/African- American	3 (3.4%)	2 (4.3%)	1 (2.4%)
Asian	1 (1.1%)	1 (2.1%)	0
Other	2 (2.2%)	1 (2.1%)	1 (2.4%)
Unknown	2 (2.2%)	2 (4.3%)	0
Mother’s Ethnicity- n (%)			
Hispanic	33 (37.1%)	17 (36.2%)	16 (38.1%)
Non-Hispanic	55 (61.8%)	29 (61.7%)	26 (61.9%)
Baby’s Ethnicity			
Hispanic	33 (37.1%)	17 (36.2%)	16 (38.1%)
Non-Hispanic	51 (57.3%)	30 (63.8%)	21 (50%)

**Table 2:** Racial and ethnic disparities in neonatal outcome clinic follow-up after PPROM and preeclampsia. *PPROM* – preterm premature rupture of membranes

	0% Attendance	1%–30% Attendance	≥ 70% Attendance
Maternal Race	30 (63.8%)	30 (63.8%)	30 (63.8%)
White/Caucasian	74 (75.5%)	30 (85.7%)	14 (87.5%)
Black/African-American	19 (19.4%)	3 (8.6%)	0
Asian	0	1 (2.9%)	0
Other	2 (2%)	0	2 (12.5%)
Unknown	2 (2%)	2 (2.7%)	0
Maternal Ethinicity	30 (63.8%)	30 (63.8%)	30 (63.8%)
Hispanic	36 (36.7%)	27 (36%)	6 (37.5%)
Non-Hispanic	62 (63.3%)	45 (61.6%)	10 (62.5%)
Percentages might not equal 100 due to missing data	30 (63.8%)	30 (63.8%)	30 (63.8%)
